# Web alert: Adaptive evolution of microbes: An annotated selection of World Wide Web sites relevant to the topics in environmental microbiology

**DOI:** 10.1111/1751-7915.14186

**Published:** 2022-12-09

**Authors:** Lawrence P. Wackett

**Affiliations:** ^1^ Department of Biochemistry, Molecular Biology & Biophysics BioTechnology Institute, University of Minnesota St. Paul MN USA

## Adaptive strategies of microbes

### https://www.frontiersin.org/articles/10.3389/fmars.2022.864797/full

This review focuses on the adaptive strategies of microbes in the oceans.

## Adaptive evolution of soil microbial communities

### https://www.pnas.org/doi/10.1073/pnas.2101254118

This study investigated how microbial composition changed spatially and temporally along a climate gradient.

## Adaptive evolution facilitated by horizontal gene transfer

### https://www.pnas.org/doi/10.1073/pnas.2007873118

This study used two closely related *Bacillus* strains and observed how positive and negative selective pressures led to gene transfers and adaption.

## Species interactions constrain adaption

### https://www.nature.com/articles/s41396‐022‐01191‐1

This study used a co‐culture of *Saccharomyces cerevisiae* and *Lactobacillus plantarum* to show that coevolution prevents the destabilization of community interactions.

## Bottlenecks in adaptive evolution dynamics

### https://www.biorxiv.org/content/10.1101/2021.12.29.474457v1

This study used modelling and empirical experiments to examine the time of adaption as a function of different inoculum sizes in the transfer regime.

## Microbial adaptive evolution and environment

### https://journals.plos.org/plosgenetics/article?id=10.1371/journal.pgen.1009314

Findings from this study suggest that changing the order of environmental changes leads to differences in adaptive and genomic changes.

## Adaptive evolution of a *Pseudomonas*

### https://sfamjournals.onlinelibrary.wiley.com/doi/full/10.1111/1751‐7915.13678

This study emphasized setting up conditions specific to a particular microorganism in adaptive evolution experiments. Specifically, they used a semi‐continuous bioreactor with a layout that prevented biofilm formation.

## Laboratory evolution to alternating substrates

### https://journals.asm.org/doi/10.1128/AEM.00410‐17

In natural environments, growth substrates are constantly changing. This study used *Escherichia coli* and alternated supplying glucose, xylose, glycerol or acetate in the growth medium.

## Design of adaptive evolution experiments

### https://journals.asm.org/doi/10.1128/AEM.03115‐16

This paper describes a model for designing and optimizing adaptive laboratory evolution experiments.

## Role of tRNAs in microbial adaption

### https://merenlab.org/2021/09/13/boats‐to‐bits/

This interesting blog post discusses the role of tRNAs in regulating protein expression and hence the ability of microbes to adapt to changing conditions in the environment.

## Extreme environments

### https://www.nature.com/articles/srep06205

This paper posits that microbial communities evolve more rapidly in extreme environments. The extreme environments examined were acid mine drainage, saline lake and hot spring and that was compared to surface ocean, freshwater, and soil.

## Laboratory natural selection

### https://faculty.washington.edu/hueyrb/pdfs/HueyRosenzweig09.pdf

This article focuses on non‐microbial systems, but it contains a wonderful description of the first documented laboratory evolution experiment with single‐celled organisms, specifically on protist adaption to increasing temperature that was conducted in the 1880's.

## Evolutionary history and microbial adaption

### https://www.osti.gov/pages/biblio/1557394

The take home message of this study is that growth rate and substrate utilization is more influenced by evolutionary history than by climate.

## Laboratory evolution of *Pseudomonas putida* KT2440 for new substrate utilization

### https://sfamjournals.onlinelibrary.wiley.com/doi/abs/10.1111/1462‐2920.14703

Wild type *Pseudomonas putida* KT2440 cannot grow exclusively on ethylene glycol but that substrate can generate reducing equivalents. Laboratory evolution developed a strain able to grow on ethylene glycol and the study revealed the nature of the mutations required for this new phenotype.

## Evolution of 4‐nitrotoluene degradation

### https://onlinelibrary.wiley.com/doi/10.1111/j.1365‐2958.2011.07817.x

While *Acidovorax* sp. strain JS42 would oxygenate 4‐nitrotoluene it would not grow on that substrate. Adaptive evolution experiments generated variants that grew on this new substrate.

## Adaptive evolution for industrial products

### https://www.ncbi.nlm.nih.gov/pmc/articles/PMC6944292/

This review article focuses on using adaptive evolution for strain construction for biotechnological applications.

